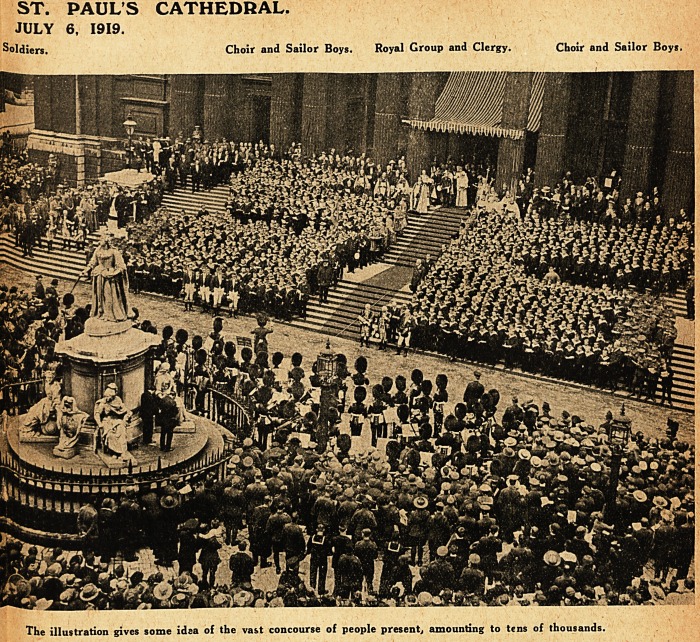# Thanksgiving for Peace

**Published:** 1919-07-12

**Authors:** 


### THANKSGIVING FOR PEACE.

THANKSGIVING SUNDAY, July 6, 1919, was a day that every inhabitant of the Metropolis will treasure in their memory and be grateful for throughout their lives, especially if it was their good fortune to be present inside or outside St. Paul's Cathedral at the Thanksgiving Services. Within the Cathedral, where the service commenced promptly at 11 o'clock, were assembled the King, wearing the uniform of an Admiral of the Fleet; and the Queen, with Queen Alexandra, who occupied chairs under the great dome. Immediately behind them were the Prince of Wales, in the uniform of the Guards, Prince Albert, in naval uniform, and Princess Mary. Other members of the Royal Family present were Princess Victoria, the Princess Royal, Princess Maud, Princess Louise (Duchess of Argyll), Princess Beatrice, the Duchess of Albany, Princess Alice, Lord Athlone, Princess Marie Louise, Lord Leopold of Mountbatten, Lord Cambridge, Lady Helena Cambridge, Lady Mary Cambridge, and Lord and Lady Carisbrooke.

Other well-known people were :

Mr. Bonar Law, Mr. Winston Churchill, Earl Curzon, Viscount Milner, Lord Ernle, Sir Auckland Geddes, Sir L. Worthington-Evans, the Lord Chancellor, Mr. E. Shortt, Mr. Austen Chamberlain, and Mr. H. W. Forster, the Earl and Countess of Courtown, Lord and Lady Aberconway, Lord and Lady Algernon Gordon-Lennox, Lord and Lady Kinnaird, Lord and Lady St. Leven, Viscount and Viscountess Clifden, Lord and Lady Saville, Lord and Lady Templemore, Viscount and Viscountess St. Davids, Marquis and Marchioness of Sligo, Lady Eva Wyndham-Quinn and Miss Burke, Earl and Countess Stanhope, the Countess of Iddesleigh, Lord and Lady Phillimore, Lord and Lady Dynevor, Marquis and Marchioness of Zetland, Lord Forester and Miss Davidson, Viscount and Viscountess Goschen, Viscount and Viscountess Galway, Lady Downham and the Hon. Rachel Florence Hayes Fisher, Lord and Lady Cochrane of Cults, Lord and Lady Hollenden, Lord Sterndale, Lord and Lady Burgh, Lord and Lady Semphill, the Earl and Countess of Harrowby and Lady Frances Rider, Viscount and Viscountess Sandhurst, Lord Joicey, Viscount and Viscountess Farquhar, Earl and Countess Bathurst, Earl Powis and Lady Hermione Herbert, the Countess of Lytton, the Hon. Mrs. Walrond, Lady Glentanar, Viscount and Viscountess Barrington, Lord Leigh, Lady Newton, Lord and Lady Stuart-Wortley, Lord Faringdon, Viscount and Viscountess Valentia, Lord and Lady Mostyn, Viscount Bryce, Viscount and Viscountess Boyne, Lord and Lady Carmichael, the Earl and Countess of Lucan, the Marquis of Aberdeen, Lord and Lady Sydenham, the Duke and Duchess of Grafton, Earl and Countess Brassey, Earl Cairns, Lady Mainwaring, Sir Charles and Lady Wade, the Hon. Mrs. Victor Cochrane, Field-Marshal Sir Douglas Haig, Admiral Sir David Beatty, Admiral Lord Beresford, Field-Marshal Lord Grenfell, Rear-Admiral Bentinck (representing the First Lord of the Admiralty), and Mrs. and Miss Bentinck; Admiral Sir Rosslyn Wemyss, General Game, Admiral the Hon. Sir Stanley Colville, Admiral the Hon. Victor Stanley, General Sir Frederick Sykes, Earl Howe, General Sir Dighton Probyn, V.C.; the Hon Sir Arthur Walsh (Master of the Ceremonies), Sir J. Follett Syng (Assistant-Master of Ceremonies), Sir Edward Wallington, the Japanese Ambassador, the Bolivian, Brazilian, Chinese, Panama, and Uruguayan Ministers, the Roumanian Charge d'Affaires, the Marchesa Imperali, Baroness Moncheur (wife of the Belgian Minister), the French Charge d'Affaires, the Secretary of the Serbian Legation.

General Sir H. H. Wilson, Lieut.-General Sir W. T. Furse, Lieut.-General Sir P. W. Chetwode, Lieut.-General Sir G. M. W. Macdonogh, Lieut.-General Sir T. E. Clarke, Major-General Sir C. H. Harington, Lieut.-General Right Hon. Sir J. G. Maxwell, General Sir H. S. Horne, Lieut.-General Sir H. de B. de Lisle, Major-General Sir F. W. B. Landon, Major-General Sir H. D. E. Parsons, Major-General Sir P. G. Twining, Major-General L. G. Blenkinsop, Major-General Sir G. P. T. Feilding, Major-General Sir P. P. de B. Radcliffe, Major-General B. Burnett-Hitchcock, Sir H. J. Creedy, Lieut.-General Sir R. E. W. Turner and Lieut.-General Sir A. W. Currie (Canada), and Lieut.-General Sir J. Monash and Lieut.-General Sir J. J. Talbot Hobbs (Australia).

Sheriff Banister Fletcher, Sheriff W. R. Smith, Sir John Bell, Sir Vezey Strong, Colonel Sir Charles Wakefield, Sir William Treloar, Colonel Sir William Dunn, Sir Edward Cooper, Mr. Alderman Briggs, Sir George Touche, Sir John Baddeley, Sir Vansittart Bowater, Sir Lulham Pound, Sir Alfred Newton, Sir Louis Newton, Mr. Alderman Moore, and many members of the Court of Common Council, with the Recorder (Sir Forrest Fulton), Chamberlain (Mr. Adrian Pollock), Remembrancer (Colonel Stuart Sankey), Town Clerk (Sir James Bell), and Chief Commissioner of Police (Sir William Nott Bower).

Representatives of the Church of Scotland and English Free Churches : Rev. Robert Kilgour, D.D. (Church of Scotland), Sir John McClure (headmaster of Mill Hill School, representing the Congregational Churches), Rev. Henry Smith (President of the Metropolitan Free Church Federation), Dr. Barber (Wesleyan Methodists); Rev. John Moore (President-designate of the United Methodist Church), Rev. M. P. Davison (Primitive Methodist Church), Commissioner Edward Higgins (Salvation Army).

Before the service began the special orchestra in the Choir played an intermezzo "Peace" for two harps and four horns, written specially for the occasion and conducted by the composer, Mr. F. Corder, and Sir Alexander Mackenzie's "Song of Thanksgiving" written on the declaration of peace after the South African War, and now repeated under the conductorship of Sir Alexander Mackenzie himself. Dr. Stanley Marchant, the sub-organist of the Cathedral, played an Idyll composed by Dr. Alan Gray, of Trinity College, Cambridge, two of whose sons were killed in the war. Their Majesties entered the Cathedral after the conclusion of the short open-air service at the West Door, and were conducted to their seats by the clergy and the Bishop of London. In front of them was an antique oak table on which rested a crimson cushion,

### THE SERVICE OUTSIDE
### PEACE SUNDAY.

Wounded

There were prayers and singing outside St. Paul's Cathedral both before and after the Thanksgiving Service within.

**Thanksgiving for Peace**—(*continued*).

Here before the service began the Lord Mayor, preceded by his sword-bearer, placed the Pearl Sword as a token of the temporary surrender of the Lord Mayor's authority in the presence of the Sovereign.

The Service began with the words: "We are assembled to praise God for the restoration of Peace, and to pray that we may worthily set forward His Kingdom of Righteousness and Peace in all the world." Then after the words "Lift up your hearts; We lift them up unto the Lord" the 103rd Psalm, "Praise the Lord, Oh my Soul" was sung, followed by the Lesson, Isaiah lxi. Then the 100th Psalm "Oh be joyful in the Lord all ye lands." The Dean then read "With our praises to Almighty God for the Restoration of Peace let us offer heartfelt thanks for the way by which during the years of war He has led us, for the bravery and devotion of our sailors, soldiers, and airmen, and for the skill, the prowess, and the patience of our merchant seamen. Let us thank Him for the unwearied work of all the men and women who have laboured to secure Victory for our Cause, for the concord in purpose and counsel between us and our Allies, and above all for the great multitude of our brethren who were faithful unto death, and whose sacrifice hallows the Peace now happily secured. And let us gather up our thanksgivings in the words of the ancient hymn wherein the Church of Christ for long ages has been wont to offer praise and adoration to God most High." Then came the Te Deum Laudamus, specially written for the occasion since the Armistice by Dr. Charles Macpherson, the Cathedral organist, who conducted the Choir and Orchestra. It was elaborate, imposing, well sung and impressive, but ceremonial. A little later the congregation joined heartily in the

### ST. PAUL'S CATHEDRAL.

### JULY 6, 1919.

**Soldiers.** **Choir and Sailor Boys.** **Royal Group and Clergy.** **Choir and Sailor Boys.**

**The illustration gives some idea of the vast concourse of people present, amounting to tens of thousands.**

**Thanksgiving for Peace**—(*continued*).

Old Hundredth, which was sung with heart and will. Then were said the following prayers:

O Almighty God, Who art a strong tower of defence unto Thy servants against the face of their enemies, we yield Thee praise and thanksgiving for deliverance from our enemies, and for Thy gracious gift of peace, we confess that it is of Thy goodness alone that we have been preserved, and we beseech Thee to continue Thy mercies towards us, that we may always acknowledge Thee as our Saviour and mighty Deliverer, through Jesus Christ our Lord.—Amen.

Almighty God, Father of all mercies, we, Thine unworthy servants, do give Thee most humble and hearty thanks for all Thy goodness and loving kindness to us and to all men, particularly to us who desire now to offer up our praises and thanksgivings for Thy mercies vouchsafed to us in the time of war and in the restoration of peace. We bless Thee for our creation, preservation, and all the blessings of this life, but, above all, for Thine inestimable love in the redemption of the world by our Lord Jesus Christ; for the means of grace and for the hope of glory. And we beseech Thee give us that due sense of all Thy mercies that our hearts may be unfeignedly thankful, and that we show forth Thy praise, not only with our lips, but in our lives, by giving up ourselves to Thy service and by walking before Thee in holiness and righteousness all our days, through Jesus Christ our Lord, to whom, with Thee and the Holy Ghost, be all honour and glory, world without end.—Amen.

Thine, O Lord, is the greatness and power, and the power and the glory, and the victory, and the majesty: for all that is in the Heaven and in the earth is Thine; Thine is the Kingdom, O Lord, and Thou art exalted as Head above all. Both riches and honour come of Thee, and Thou rulest over all; and in Thine hand is power and might, and in Thine hand it is to make great and to give strength unto all. Now, therefore, our God, we thank Thee, and praise Thy glorious Name.

---

Then the Dean said: "Now unto the King Eternal, Immortal, Invisible, the only wise God, be honour and glory for ever and even. Amen."

**Thanksgiving for Peace**—(*continued*).

### The Archbishop of Canterbury's Address.

Then came the Address, the Archbishop said:—

We are met at a great hour in the world's life. It is for an intensely solemn purpose. Our service to-day stands out by itself as commemorating what is literally the greatest event in human history. Many a time in our long island story the men and women of England have gathered here to give thanks for victory and peace. The Armada, Blenheim, Waterloo, Sebastopol give examples of such occasions. The very first service to which these actual walls, then fresh and white from the mason's chisel, gave echo was the thanksgiving for a famous peace. But never, never till to-day have King and Queen and princes come hither to give thanks, along with Lords and Commons, with Navy and Army and Airmen, with statesmen and governors from the King's Dominions overseas, with kinsmen from the great Republic of the West, with Ambassadors from friendly States, and, newest of all, with banded companies of men and women workers enrolled for active ministries of war or peace.

That answers to the vastness of the hour. And to-day this gathered multitude stands together, sings together, kneels together: for what? Not just in order to say how we rejoice that the mightiest of all wars is ended, that the peril is rolled away, and that victory is won, but in order, as members of Christ's society on earth, Churchmen and Nonconformists side by side, to give definite, thoughtful, loyal recognition to the Lord God Almighty for what He has done for us in the years of war, and their issue in the victorious peace. The Lord God Omnipotent reigneth. "I was glad when they said unto me, Let us go into the house of the Lord."

We need no preacher's words to-day. Our hearts are full. The vision sets them aflame. Look back. Look round. Look onward.

On this very day one year ago King and Queen were kneeling where they kneel this morning, and the arches overhead were filled with our chanted thanks to God for twenty-five completed years of wedded life and love and leadership. And while we were kneeling here the guns were thundering on the Western battle-front; armies were rolling up for the second battle of the Somme, and in Italy and the East the banks and plains of the Piave and the Jordan and the Tigris were still alive with war.

Carry your thoughts a year further back to a more local and passing thing. It was on the morrow of this day two years ago that there fell on us the disquiet of the great daylight air-raid upon London, in vivid contrast with our jubilant streets to-day. Back yet another year. It was this opening July week in 1916, three years ago, which has given imperishable fame to woods and villages in what were once the lovely valleys of the Somme: Mametz, Thiepval, Contalmaison, and the rest. Yet one more memory. It was in the torrid weeks of an Ægean July just four years ago that the world's records of heroism were being enriched by what Gallipoli can tell.

It is by that backward look along the years that we appraise aright the spirit of thanksgiving which is rightly ours to-day. Not merely—to some of us not mainly—thanksgiving that it is over. We are proudly thankful not only for the peace which has been won, but for the price at which we won it. The glad self-sacrifice of those among our best in power and promise whose young, strong, eager lives were unhesitatingly given for our country's honour and for the bettering of the world is an enduring asset in the treasure-house of what we reverently hold most dear. And not less the offering of those whose buoyant manhood has been scarred and marred by what war has wrought, and whom, present with us still, we reverence for what they too have done for us all.

Our gaze passes from what has happened to what is happening, or is round about us, now. What do we see as the guerdon which our brave men have won for us? We call it peace. And peace means, not simply the ending of strife, but the spirit, the conditions in which whatsoever things are just and pure and wholesome can flourish and abound. Is that what we have won? If not, there is something amiss, something which needs fashioning still. But, please God, what we have secured by these years of unutterable stress is and shall be just that. Whole-heartedly we mean it so to be for our land and for other lands as well.

I stand here, I speak here, to-day as one who, believing in our Master's promise, is bold to maintain—despite all our qualms; despite, nay because of, our experience—that, in His good time, the ending of war between Christian peoples is a thing attainable. Slow and halting are our steps upon His way, but the victories of Jesus Christ among the sons of men are manifold, are visible, are proven well. The world, with all its wrongs, is better than it was. Bit by bit its evils wane. May it perhaps be that in the very horribleness of these five hideous years we have seen as of old that the evil spirit can tear its victim before it be cast out? "Peace, be still." To that vision, as yet dim and hazy and uncertain, our eyes turn as we look forward wistfully into the unborn years. It is still a thing unfashioned. But in our prayers at least it has its firm place. "Thy kingdom come." Does anyone as he offers that prayer—our Master's prayer—mean a kingdom among men wherein war is still to be the arbiter? And, if not? If not, it depends on those who pray—"Thy kingdom come": for "The Kingdom of God is within you."

And so, along with prayer and vision, there comes effort—clear, sustained, robust, believing. To that resolve, that effort, we have as a people set our hand. A League of Nations must be no mere theory of statesmen. It is to be the peoples' pact. So far as in us lies we are answerable before God and man that it live and grow; and the people—you and I, that is—must be worthy to be its artificers. A people of clean life, of sensitive honour between man and man, of ready recognition of "the other man's" side: a people keen at home in mutual service, and therefore strong in contribution to the common pact among the nations of the world.

Now all that is not going to come about of a sudden. "If the vision tarry, wait for it." Bethlehem brought a new message to mankind.

> "Beneath the angel-strain have rolled
> Two thousand years of wrong."

But Christendom mayhap is still young as compared with what its life shall be. With chastened and yet eager heart we are thanking God to-day for what these five years have brought us, for the trust of championship on behalf of what is just and of good report, for the ready self-offering of our worthiest, for the dauntless valour of their gift, for the intrepidity and resource of our high command. For the victory that has been won.

And for the achievement of civilian leaders, too. The

**Thanksgiving for Peace**—(*continued*).

nobly persistent toil of statesmen who, through tangles dense enough to daunt the stoutest heart, have wrought and tramped and even hewed their way to an outlet, or towards an outlet, a pathway of permanent peace. The pathway may be rugged still. It may want, I think it will want, consideration and adjustment here and there as the months or years run on. But it is achieved, and we can go forward in thankfulness and hope to the tasks which lie immediately ahead. Outstanding, surely, among these is the task of staying, throughout Europe, if we may, of one of the darkest ravages of war, the scourge of impending famine. Great tracts, we are told, are in want of daily bread. The obligation rests upon us all, as a nation and as men.

We start with the new joy of a fellowship widened and deepened by the stern discipline of these strenuous years. The bonds are strong. Some of them are new. We shall need them all if we are to stand together aright—

"To find, to fashion, and fulfil
The cleaner life, the sterner code."

We have won the peace for which we strove. We thank God for it here and now. May He give us, as He only can, the grace to use it worthily. We kneel together to-day, King and people, in fresh dedication of ourselves as a nation to the service of the Lord Christ. It is not mere aspiration, mere feeling, that we want, but firm unflinching will—

"We know the paths wherein our feet should press,
Across our hearts are written Thy decrees;
Yet now, O Lord, be merciful to bless
With more than these.

"Courage we ask not—knowledge Thou hast lent,
But Lord, the will—there lies our bitter need,
Give us to build above the deep intent
The deed, the deed."

At the conclusion of the Address the congregation sang Hymn 379,

"Now thank we all our God,
With heart, and hands, and voices,
Who wondrous things hath done,
In Whom the world rejoices;
Who from our Mother's arms
Hath blessed us on our way
With countless gifts of love
And still is ours to-day."

Then the Archbishop, standing in the pulpit, read the following prayers:—

Brethren, we have now with unfeigned hearts made an offering of praise and thanksgiving to the Lord our God for the peace wherewith the long struggle of the past years has been crowned. Yet we must needs remember the many and grave anxieties by which we are still encompassed. The peace has to be made stable and secure. In many nations tumult and disorder are rife, and the people are in sore distress. In our own land we have varied and conflicting difficulties to overcome in our determined effort to build a new and better order in our common life. Wherefore it is meet that we should now join together in humble prayer to Almighty God that as He has thus far prospered us so He would continue His grace and mercy towards us, delivering us from the perils which still surround us, and leading us by His Holy Spirit into a lasting fellowship of all classes and nations. And let us ask Him that we may be enabled to seek first His Kingdom and righteousness, and may be found worthy to have added unto us all those things which we desire for the good of our people and of all mankind.

O Lord, hear our prayer:
*And let our cry come unto Thee.*

Let us pray that Peace may be true and lasting.

Almighty God, from whom all thoughts of truth and peace proceed, kindle, we pray Thee, in the hearts of all men the true love of peace, and guide with Thy pure and peaceable wisdom those who take counsel for the nations of the earth; that in tranquillity Thy Kingdom may go forward, till the earth is filled with the knowledge of Thy love; through Jesus Christ our Lord. Amen.

Let us pray for union and concord within the nations.

O Eternal God, our heavenly Father, Who alone makest men to be of one mind in a house, we pray Thee to appease the tumults and violence which disturb the nations, and in all countries to give concord and goodwill; most humbly beseeching Thee to grant to all of us grace, that we may henceforth obediently walk in Thy holy commandments; and, leading a quiet and peaceable life in all godliness and honesty, may continually offer unto Thee our sacrifice of praise and thanksgiving for Thy mercies towards us; through Jesus Christ our Lord. Amen.

Let us pray for the League of Nations.

O Almighty God, Who canst bring good out of evil, and makest even the wrath of man to turn to Thy praise, teach Thy children to live together in charity and peace; and grant, we beseech Thee, that the nations of the world may henceforth be united in a firmer fellowship for the promotion of Thy glory and the good of all mankind; through Jesus Christ our Lord. Amen.

Let us pray for the Church.

O Lord Jesus Christ, Who didst say to Thine Apostles, Peace I leave with you, My peace I give unto you, regard not our sins, but the faith of Thy Church, and grant us that peace and unity which is agreeable to Thy will; Who livest and reignest with the Father, and the Holy Spirit, one God, world without end. Amen.

Let us pray for rulers and for those in authority.

Almighty God, Who alone givest wisdom and understanding, inspire, we pray Thee, the hearts of all to whom Thou hast committed the responsibility of government in the nations of the world. Give to them the vision of truth and justice, guide them to know how best to temper justice with mercy, that by their counsels the nations may work together in true brotherhood, and Thy Church throughout the world may serve Thee in unity and peace; through Jesus Christ our Lord. Amen.

Let us pray for the British Empire.

O Lord God of our fathers Who in Thy goodness hast led this people hitherto by wondrous ways, Who makest the nations to praise Thee, and knittest them together in the bonds of peace; We beseech Thee to pour Thy blessing on the Empire over which Thou hast called Thy servant George to be King. Grant that all, of whatever race or tongue, may, in prosperity and peace, be united in the bond of brotherhood, and in the one fellowship of the Faith, so that we may be found a people acceptable unto Thee; through Jesus Christ our Lord. Amen.

Let us pray for all who have suffered through the war.

Unto Thy loving kindness, O Lord, we commend all those who are stricken and suffering by reason of the

**Thanksgiving for Peace**—(*continued*).

war: the wounded and the overstrained, the disabled, the blinded, the homeless, and the oppressed; all who have been bereaved of those dear to them; all whose faith in Thee has been shaken by what they have seen or suffered. Strengthen them, O God, with Thy Holy Spirit, and give them courage and hope. Help us to do our part in ministering to them; for the sake of Him who bore for us the pain and desolation of the Cross, Thy Son our Saviour Jesus Christ. Amen.

Let us commend to the mercy of God those who have fallen in the service of their country.

O God of the spirits of all flesh, we praise and magnify Thy Holy Name for all Thy servants who, having fought a good fight, have finished their course in Thy faith and fear; and we beseech Thee that, encouraged by their examples and strengthened by their fellowship, we with them may be found meet to be partakers of the inheritance of the Saints in light; Through the merits of Thy Son Jesus Christ our Lord. Amen.

Let us dedicate ourselves anew to the service of Christ.

O Lord Christ, Thou Prince of Peace, The Faithful and True, Who in righteousness dost judge and make war; Grant to us all, we beseech Thee, that, putting on the whole armour of God, we may follow Thee as thou goest forth conquering and to conquer; and, fighting manfully under Thy banner against sin, the world, and the devil, we may be found more than conquerors, and at the last may be refreshed with the multitude of peace in the heavenly Jerusalem, the holy City of our God; Whose is the greatness and the power, the victory and the majesty, for ever. Amen.

Let us sum up our prayers and praises in the words which our Lord Himself has taught us, saying:

Our Father, which art in heaven, Hallowed be Thy Name, Thy kingdom come, Thy will be done, in earth as it is in heaven. Give us this day our daily bread, And forgive us our trespasses, As we forgive them that trespass against us. And lead us not into temptation: but deliver us from evil: For Thine is the kingdom, the power, and the glory, For ever and ever. Amen.

THE BLESSING.

Unto God's gracious mercy and protection we commit you; The Lord bless you and keep you. The Lord make his face to shine upon you, and be gracious unto you. The Lord lift up the light of His countenance upon you, and give you peace, both now and evermore. Amen.

The singing of "God save the King" concluded the service, and led by the Bishop of London and the clergy Their Majesties and the members of the Royal Family, with others of the congregation from under the Dome, returned to the West Door. As the King and Queen left the Cathedral trumpeters of the Royal Horse Guards played a fanfare from the West Gallery. Standing in the centre of the West Door, with the Royal Family grouped around him, "God save the King" was sung by the vast audience, amounting to tens of thousands of people, with two new verses, which we annex. The singing was most hearty and inspiriting, and could be heard for a considerable distance, being most impressive and striking. Those who heard it are not likely to forget it.

**The New Verses to the National Anthem.**

One realm of races four,
Blest more and ever more,
God save our land!
Home of the brave and free,
Set in the silver sea,
True nurse of chivalry,
God save our land!

Kinsfolk in love and birth,
From utmost ends of earth,
God save us all!
Bid strife and hatred cease,
Bid hope and joy increase,
Spread universal peace,
God save us all!

**Homage and Worship.**

Under this heading *The Times* reports:—

The knowledge that the King and Queen, with their children, were to attend St. Paul's Cathedral brought a great crowd into the City yesterday morning. The streets of the old City converging upon the Cathedral, usually so quiet and deserted on a Sunday, presented from an early hour a scene of unwonted animation.

The sky was dull and the air chilly, with some threat of rain, but this was no ordinary occasion, and the weather did not matter. The gathering of the people was a national act of homage and of worship. Not a tithe of the people, it is true, could hope to enter the Cathedral or even to approach its steps, and not a tithe expected to do so. Their hope and their desire, by their mere presence somewhere on the route of the Royal progress, was to associate themselves in heart and in mind with their King and Queen in a humble act of piety on a great and memorable occasion in our national history. In that sense, the whole vast gathering of people in the streets was part of the congregation inside the Cathedral, and their whole bearing and demeanour proclaimed it.

As in the City itself, so it was along the whole route, from the Palace to Charing Cross, past the Strand, down Fleet Street, and up Ludgate Hill to the Cathedral; through New Bridge Street, by Blackfriars, and along the Thames Embankment on the way back—everywhere, on both sides of the road, the people, three and four deep, men, women, and children, dressed in their Sunday best, quietly awaited the arrival of their King and Queen. Above them floated the flags of Old England, of the Empire and its sister nations, and of the Allies. As the familiar scarlet of the Royal livery came into sight, cheers filled the air, hats were waved, and handkerchiefs fluttered. The King and Queen, with whom was the Prince of Wales, repeatedly acknowledged these manifestations of loyalty. In the second carriage was Princess Mary, with her younger brother, Prince Albert. The popularity of the Princess was shown by the renewed bursts of cheering which greeted her, and very gracefully she bowed in acknowledgment again and again.

The cheering was hearty and full of exultant feeling, but never boisterous. The people were cheerful anl happy, but calm and subdued, as befitted the occasion, for the dominant note of the whole Royal progress, from the moment the King's carriages set out from Buckingham Palace until they returned, was that of the great religious thanksgiving in which it culminated in St. Paul's Cathedral. That was the feeling which inspired everybody. "'Tis a great occasion, lad," exclaimed one old lady who had come to pay homage to her King, speaking to her little grandson; "'tis a great occasion, such as you and I have never seen before, nor ever shall again."

## Figures and Tables

**Figure f1:**
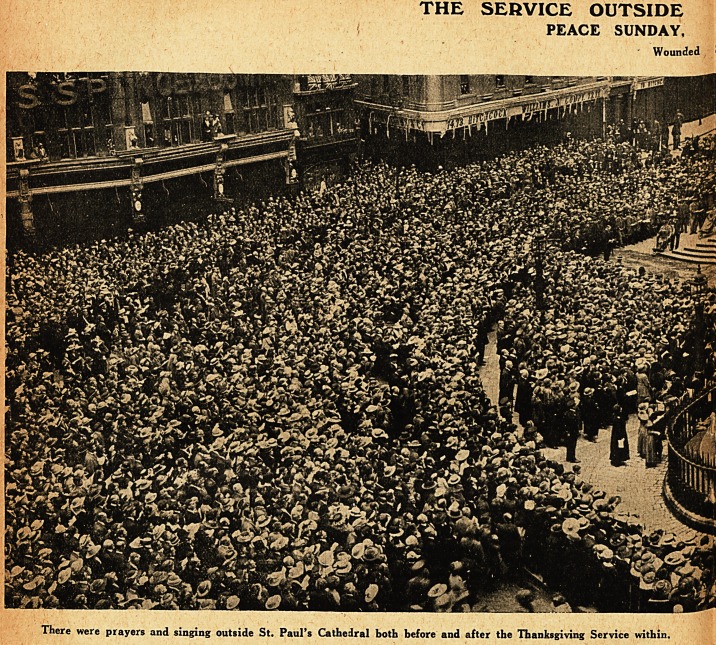


**Figure f2:**